# Cardiac Output Monitoring by Pulse Contour Analysis, the Technical Basics of Less-Invasive Techniques

**DOI:** 10.3389/fmed.2018.00064

**Published:** 2018-03-06

**Authors:** Jörn Grensemann

**Affiliations:** ^1^Department of Intensive Care Medicine, Center of Anesthesiology and Intensive Care Medicine, University Medical Center Hamburg-Eppendorf, Hamburg, Germany

**Keywords:** hemodynamics, cardiac output, monitoring, physiologic, pulse contour analyses, waveform analysis, arterial wave property

## Abstract

Routine use of cardiac output (CO) monitoring became available with the introduction of the pulmonary artery catheter into clinical practice. Since then, several systems have been developed that allow for a less-invasive CO monitoring. The so-called “non-calibrated pulse contour systems” (PCS) estimate CO based on pulse contour analysis of the arterial waveform, as determined by means of an arterial catheter without additional calibration. The transformation of the arterial waveform signal as a pressure measurement to a CO as a volume per time parameter requires a concise knowledge of the dynamic characteristics of the arterial vasculature. These characteristics cannot be measured non-invasively and must be estimated. Of the four commercially available systems, three use internal databases or nomograms based on patients’ demographic parameters and one uses a complex calculation to derive the necessary parameters from small oscillations of the arterial waveform that change with altered arterial dynamic characteristics. The operator must ensure that the arterial waveform is neither over- nor under-dampened. A fast-flush test of the catheter–transducer system allows for the evaluation of the dynamic response characteristics of the system and its dampening characteristics. Limitations to PCS must be acknowledged, i.e., in intra-aortic balloon-pump therapy or in states of low- or high-systemic vascular resistance where the accuracy is limited. Nevertheless, it has been shown that a perioperative algorithm-based use of PCS may reduce complications. When considering the method of operation and the limitations, the PCS are a helpful component in the armamentarium of the critical care physician.

## Introduction

Critically ill patients often receive extended hemodynamic monitoring with measurement or estimation of cardiac output (CO) as an aid for guiding fluid and vasopressor therapy. With the introduction of the pulmonary artery catheter by Swan et al., measurement of CO became available at the bedside by a thermodilution technique (1). However, routine use of the pulmonary artery catheter in critically ill patients has been questioned, presumably owing to its invasiveness, requiring an additional venous access with a dedicated catheter inserted into the pulmonary artery (2) and due to difficulties in the interpretation of the results (3).

In the following years, transpulmonary thermodilution techniques have been introduced, still allowing for a measurement of CO while less invasive, only requiring a central venous and an arterial line that are often used for standard hemodynamic monitoring in intensive care patients (4). However, the femoral access for the arterial line is preferred since the tip of the dedicated thermistor catheter must be placed in a central artery for correct measurements.

To further reduce the invasiveness of CO measurements, several new techniques have been evaluated and introduced into clinical practice in recent years. These include “non-invasive methods” and “less- or minimal-invasive methods.”

The non-invasive methods estimate a CO, i.e., from changes in thoracic electrical impedance, from radial applanation tonometry (T-Line), or from finger blood pressure cuffs (Nexfin™/Clearsight™, CNAP-Systems).

The less- or minimal-invasive methods estimate a CO from an arterial pulse contour waveform (5–7) and require only a conventional arterial line to obtain an input signal. Although some systems may be calibrated by manually entering a CO measured with an independent reference technique (i.e., echocardiography), they do not include an independent calibration method and are thus also referred to as “uncalibrated pulse contour methods.” Currently, four systems of this type have been introduced into clinical practice (see Table 1).

**Table 1:** Overview of uncalibrated pulse contour systems.

System	Distributor	Method	External calibration	Requirements
FloTrac™/Vigileo™^a^	Edwards LifeSciences, Irvine, CA, USA	Sampling at 100 Hz, multiplication of pulse rate with SD of arterial pressure and a conversion factor	No	Dedicated transducer

LiDCOrapid™	Medtronic, Minneapolis, MN, USA	PulseCO™ algorithm, waveform independent pulse power analysis	Yes—external cardiac output (CO) input	Keycard^b^

ProAQT™/Pulsioflex™	PULSION Medical Systems, Feldkirchen, Germany	Sampling at 250 Hz, area under curve of the systolic portion of waveform multiplied by calibration factor	Yes—external CO input	Dedicated transducer

PRAM™/MostCare™	Vytech, Padova, Italy	Pressure recording analytical method, sampling at 1,000 Hz, calculation from perturbations	No	Keycard^b^

*^a^Distribution of the Vigileo™ monitor has been discontinued, the FloTrac™ method is available in the EV1000™ monitor that also includes the transpulmonary thermodilution technique*.

*^b^A keycard is required for operation, on which either a number of booked applications or an unlimited activation code is encoded*.

In addition, some systems have been introduced that combine a pulse contour analysis with an internal reference method and are referred to as “calibrated pulse contour methods.” Two transpulmonary thermodilution systems are available that track CO by pulse contour analysis after an initial calibration with algorithms, which are similar to the ones used in the stand-alone uncalibrated PCS (PiCCO_2_™: similar to Pulsioflex™, PULSION Medical Systems, Feldkirchen, Germany; EV1000™: similar to Vigileo™, Edwards LifeSciences, CA, USA). The algorithm of the LiDCOrapid™ is also used in the LiDCOplus™ System (Medtronic, MN, USA) that can measure CO by a lithium dilution method. Furthermore, a pulse contour analysis is available, that is calibrated by an esophageal Doppler included in the same monitor (CardioQ-ODM+, Deltex Medical, Chichester, UK). The use of the pulse contour analysis without the Doppler calibration is not possible.

The aim of this review is to focus on the technical basics of uncalibrated pulse contour methods for monitoring of CO.

## Basic Considerations

The uncalibrated PCS are designed to be connected to a radial or a femoral arterial catheter. CO is estimated continuously after an optional external calibration possible with some systems. Basically, all systems calculate CO by multiplying stroke volume (SV) and heart rate. Heart rate is usually equal to the pulse rate. The input of the device consists of a pressure measurement, the arterial waveform. Obtaining the pulse rate and hence the heart rate from the waveform is usually a straight-forward task by counting the number of upstrokes of the pressure curve over time. The calculation of the other required parameter, the SV is a difficult task since a pressure measurement must be converted to a volume measurement. This is done by estimating flow that is integrated over time leading to a volume. Deriving a flow from a pressure parameter requires concise information of the pressure–volume relation in the arterial system and especially the aorta. The systems use a refinement of Otto Franks Windkessel model dating back to 1899 that incorporates arterial impedance (Za), arterial compliance (Ca), and systemic vascular resistance (SVR) (8).

Arterial impedance is the ratio of pressure to flow in the central arteries and is determined by the physical properties of the arterial walls. It represents the forces opposing the propagation of the pressure wave transmitted along the arterial system. The arterial compliance is defined as the difference of blood volume induced by a difference in pressure and mainly depends on the elastic properties of the arterial walls. The SVR is the resistance of the total systemic vasculature to the blood flow.

Since arterial impedance and arterial compliance cannot be measured non-invasively, all PCS must obtain a good estimate of these parameters. All systems except one use internal nomograms or databases based on demographic data, i.e., age and gender.

## Operating Principles of the PCS

### Vigileo™

The Vigileo™ monitor uses the proprietary FloTrac™ transducer that is attached to a standard radial or femoral arterial catheter. No external calibration is required. This system samples the arterial waveform at 100 Hz and then determines CO in 20 s intervals by a multiplication of the pulse rate with the SD of the arterial pressure over a certain period and a so-called “conversion factor” χ. This factor corresponds to the vascular tone and is calculated by means of a multivariate polynomial function. This function includes pulse rate, body surface area, aortic compliance, mean arterial pressure, and the SD of the arterial pressure over a certain time, as well as skewness and kurtosis of the waveform which describe the form of the arterial pressure curve. The aortic compliance is determined from an internal demographic data base (age, sex, height, and weight) and mean arterial pressure.

Over the years, the FloTrac™ algorithm has been refined. In the current software version 4.0, the internal database has been expanded, and an improved SV tracking has been added, electronically eliminating, and interpolating abnormal beats, i.e., in premature complexes.

### Pulsioflex™

The Pulsioflex™ monitor is connected to the proprietary ProAQT™ transducer attached to a standard radial or femoral arterial catheter. A start CO may be determined by two methods. A CO may be manually entered if available from an external calibration method, i.e., echocardiography. Alternatively, the monitor may be “autocalibrated” thus estimating a CO from an internal database based on patient’s characteristics (age, body height and weight, and gender).

For the continuous measurement of CO, the arterial waveform is sampled at a frequency of 250 Hz. The systolic portion of the arterial waveform is identified, and the area under the curve (AUC) integrated from pressure over time. At the start of CO measurements, an internal calibration factor is calculated from the CO and AUC. Since the AUC is proportional to CO, an increase of the AUC corresponds to an increase of CO, and a decrease of AUC corresponds to a decrease of CO. The algorithm also takes SVR and arterial compliance into account to improve measurements. The internal calibration factor basically corresponds to the arterial impedance.

### LiDCOrapid™

This system is connected to any arterial line without a dedicated catheter. No special pressure transducer is required; the monitor can receive the pressure signal from a conventional vital signs monitor *via* an analog output. The system can be calibrated by manually entering a CO from a reference method.

The algorithm of this system for determining CO relies on calculation of SV by means of a so-called “pulse power analysis,” which is independent of the shape of the arterial pressure curve. This algorithm determines a nominal aortic volume from a monitor-internal nomogram, in which age, gender, height, and other parameters are considered. The obtained volume is multiplied by an exponential function, that is affected by arterial blood pressure and an aortic compliance determined from an internal reference.

### MostCare™

This system uses an algorithm called “pressure recording analytical method” (PRAM). The approach of this system is different from the other methods because the arterial impedance that is required for calculation of SV is estimated from perturbations of the arterial pressure waveform and not derived from internal nomograms based on demographic parameters (5). No external calibration is available with this system. The system does not require a dedicated pressure transducer.

The estimation of the arterial impedance relies on a complex theory of perturbations and is obtained from a morphological analysis of the pulsatile and the continuous components of the pressure waveform (9). When the pressure wave is propagated through the arterial system, the opposing force generated by the specific arterial impedance of the vasculature reflects parts of the pulse wave. The reflection leads to small oscillations (“perturbations”) in the pulse wave that are recorded with a sampling frequency of 1,000 Hz. An increase of the impedance leads to a consecutive increase in the perturbations. These perturbations are obtained separately for the systolic and the diastolic part of the pulse wave. An arterial impedance is then estimated from the magnitude and the difference of systolic and diastolic of the perturbations.

Further calculations for SV use the area under the systolic part of the pressure curve divided by the arterial impedance. CO is obtained by the multiplication of pulse rate and SV.

## Prerequisites for Monitoring

A good arterial waveform signal is an important prerequisite for correct measurements. Over-dampened and under-dampened waveforms indicate that the dynamic response characteristics of the arterial catheter system are insufficient and therefore not suitable for analysis (10). It has been estimated that approximately 30% of arterial waveforms in intensive care units are either over- or under-dampened (11). The PCS do not incorporate an automatic detection for inappropriate waveform readings thus requiring the operator to visually inspect and evaluate the arterial waveform regularly and confirm that the signal is correct.

A rapid flush test has been proposed to analyze the intrinsic resonance frequency of the catheter–transducer system and to verify for correct response characteristics (12, 13). After termination of the square wave from the rapid flush no visible oscillations of the wave indicate overdamping while several oscillations and “ringing” point to underdamping (14). The resonance frequency of the catheter–transducer system can be measured, and the damping coefficient calculated for a precise evaluation of the response characteristics that can be compared with the nomogram by Gardner (10). As a rule of thumb, an adequate response indicating appropriate dynamic response characteristics can be expected when the waveform returns to the pulse waveform after one to three undulations after the rapid flush test. The dynamic response characteristics change over time but may be corrected by a rapid flush (15). However, this requires a regular intervention by the operator.

Most systems rely on the recognition of the dicrotic notch to identify the systolic portion of the pulse waveform. In instances of over- or under-dampened catheter–transducer systems, the dicrotic notch may not be identified correctly, possibly leading to incorrect measurements (16).

Although these systems are designed to be used with any arterial waveform, concerns have been raised that femoral and radial waveforms are not interchangeable (17, 18). Trending analysis for one of the systems has been shown to be superior when the device was connected to a femoral catheter over a radial catheter (19).

## Limitations to Pulse Contour Monitoring

As outlined earlier, the minimally invasive pulse contour analyses systems must convert a pressure measurement into a volume parameter. Since it is impossible to non-invasively measure the determinants needed for this transformation, this transformation is prone to error, especially during some underlying pathologies.

### Systemic Vascular Resistance

As data show, agreement of the minimal-invasive methods with reference methods is poor, especially in patients during low SVR states such as sepsis and chronic liver failure (20–22). Apparently, there are no specific morphological patterns in the arterial waveform that are pathognomonic for low SVR. The difference of radial and central arterial pressure increases in low SVR (17), presumably reducing the ability of the systems to estimate adequate CO values. It has been shown that pulse contour monitoring systems are vulnerable in states of SVR changes, i.e., vasopressor therapy (21, 23). However, there are differences between the systems and some seem more robust than others (24).

The MostCare™ system that derives the arterial impedance from the pressure waveform itself and not from an internal nomogram overestimates CO in low SVR states (25), but data are sparse so far, and further validation in these patients is warranted.

The manufacturer of the LiDCOrapid™ system does not recommend its use during peripheral vasoconstriction.

### Intra-aortic Balloon Pump

Due to the altered arterial waveform during intra-aortic balloon-pump therapy, most systems cannot identify the systolic and diastolic portion of the waveform correctly. Therefore, these systems are not able to display correct values. The use of the LiDCOrapid™, Vigileo™, and Pulsioflex™ systems is not recommended by the manufacturers. For the MostCare™ system, a study with 15 patients could show a good accuracy in patients with IABP, probably due to its different approach to measurements (26).

## Accuracy and Trending Ability

The accuracy and trending ability are used for the evaluation of the pulse contour analysis systems. While accuracy is the agreement with an absolute value of a reference method, trending ability indicates the extent to which a change in CO over time is correctly estimated. As described earlier, none of the methods perform a measurement. Since the CO is only estimated according to different algorithms, the pulse contour analysis methods are error prone when used in critically ill patients who often have a low SVR, i.e., in septic shock as outlined earlier.

The Vigileo/FloTrac™ algorithm has been refined several times, and the manufacturer claimed an improvement in accuracy and trending ability with each iteration of the software. However, the available validation studies for the up to date fourth generation software could not show a sufficient accuracy or trending ability for this system when used in patients with changes in SVR or with low CO, although the performance has improved over the previous software versions (27–29). Concerning the Pulsioflex/ProAQT™ system data show that this system is also unable to adequately estimate the CO during low- or high-SVR. Concerning the trending ability, data are ambiguous, and the performance of this system may rely on the arterial access. The performance seems better when the system is connected to a femoral arterial catheter than to a radial arterial catheter (19, 21, 22). Similar to the other systems tested, accuracy of the LiDCOrapid™ monitor is below the acceptable limits (30). At first, validation studies of the MostCare/PRAM™ algorithm have shown a good agreement with thermodilution as reference method (31–33), followed by studies that have shown that accuracy is below acceptable limits (34, 35). This method should therefore be further evaluated.

In summary, none of the systems has a sufficient accuracy to be used in critically ill patients. Nevertheless, it must be noted that despite the inability to correctly estimate CO, it has been shown that these systems may improve outcome when used intraoperatively and algorithm based (36–39), although one large randomized study in high-risk abdominal surgery found no benefit (40).

Therefore, it is important that the pulse contour methods are used in a targeted manner in selected patients, where a benefit for their application could be shown. In critically ill patients or if accuracy plays a role, transpulmonary or pulmonary artery thermodilution methods should be used.

## Conclusion

Uncalibrated PCS are less-invasive methods to estimate CO. Only a conventional arterial catheter that is present in many critically ill patients is required. For the transformation of the arterial waveform as a pressure signal to CO, assumptions on the dynamic characteristics of the arterial vasculature must be made. Most systems use internal databases or nomograms based on demographics, while one system uses a complex calculation to estimate the necessary parameters.

Special attention has to be given to the arterial waveform as the input signal of the pulse contour monitors. Neither over- nor under-dampened signals are suitable for analysis and regularly require the operator’s intervention for the assessment of the dynamic response characteristics of the catheter–transducer system, i.e., by a fast-flush test. The operator must confirm that the waveform is correct before obtaining CO values from the monitor.

The use of PCS is limited in patients with large deviations from normal SVR or in patients receiving intra-aortic balloon pumps. The accuracy and trending ability of the CO estimation compared with thermodilution measurements is often limited. However, it has been shown that an algorithm-based use of PCS can improve the perioperative outcome of patients. Uncalibrated PCS represent a helpful, less-invasive tool in the hemodynamic armamentarium of the critical care physician.

## Author Contributions

The author listed has made a substantial, direct, and intellectual contribution to the work and approved it for publication.

## Conflict of Interest Statement

The author declares that the research was conducted in the absence of any commercial or financial relationships that could be construed as a potential conflict of interest.
